# Supplementary material for: Localised surface plasmon resonance inducing cooperative Jahn–Teller effect for crystal phase-change in a nanocrystal
Source: Nat Commun. 2023 Jul 31;14:4471. doi: 10.1038/s41467-023-40153-9 (PMC10390505; doi:10.1038/s41467-023-40153-9)
Supplement: Supplementary file 1 — Supplementary Information [file 41467_2023_40153_MOESM1_ESM.pdf]

Supplementary Information

Localised surface plasmon resonance inducing cooperative Jahn–Teller effect for crystal phase-change in a nanocrystal

Masanori Sakamoto[1], Masaki Hada[2]*, Wataru Ota[3,4], Fumihiko Uesugi[5], & Tohru Sato[3,4]*

[1] Institute for Chemical Research, Kyoto University, Uji, Kyoto 611-0011, Japan.
[2] Tsukuba Research Center for Energy Materials Science (TREMS), Faculty of Pure and Applied Sciences, University of Tsukuba, 1-1-1 Tennodai, Tsukuba 305-8573, Japan.
[3] Fukui Institute for Fundamental Chemistry, Kyoto University, Sakyo-ku, Kyoto 606-8103, Japan.
[4] Department of Molecular Engineering, Graduate School of Engineering, Kyoto University, Nishikyo-ku, Kyoto 615-8510, Japan.
[5] National Institute for Materials Science (NIMS), 1-1 Namiki, Tsukuba, Ibaraki 305-0044, Japan.

Materials

All solvents and reagents were used as received without further purification. Copper(I) acetate ($(CH_3COO)_2Cu$, 97%) and 1-octadecene (90 %) were purchased from Sigma Aldrich Ltd. Oleylamine (OAm, C18 content 80–90%) was purchased from Acros Organics and distilled before use. Sulphur powder (S, > 99%) was purchased from KOUJUNDO CHEMICAL Laboratory Co., Ltd.

Characterisation

A JEM-2200FS (JEOL) electron microscope with an operating voltage of 200 kV was used for high-resolution transmission electron microscopy (HRTEM), while XRD patterns were recorded on a PANalytical Aeris diffractometer with Cu Kα radiation (λ = 1.542 Å) at 40 kV and 15 mA. Ultraviolet–

visible–near-infrared (UV–Vis–NIR) absorption spectra were recorded using a UV-3600 spectrophotometer (Shimadzu). The temperature-dependent resistivity was measured using the van der Pauw method and a ResiTest8400 (TOYO Corporation) equipped with a cryostat.

Sample preparation and TEM observations

A suspension containing CuS and ethanol was dropped onto a carbon-mesh grid to fabricate the TEM sample. TEM images and diffraction patterns were acquired using Cs-corrected TEM with a cold field-emission gun (JEOL JEM-ARM200F) and an accelerated voltage of 200 keV. A light-irradiation TEM specimen holder was used for observations with and without light irradiation. The specimen holder loaded the specimen into the microscope column containing a thin SiO_2 rod and mirror in its head, an optical fibre connected the 170-mW Xe lamp (PE300BF, Cermax). UV–Vis light could reach the specimen through the rod reflected by the mirror.[S1] Since the efficiency of reflection of the mirror is approximately 80%, the power of light irradiated on the sample was approximately 140 mW/cm^2.

Simulation procedure

Diffraction patterns were calculated using the abtem[S2] library of Python. The thickness of the sample was set to 50 Å during calculations, and the incident beam direction was [0001]. The STEM mode with a small convergence angle was used, and only the Cu atoms were assumed to exhibit movement (by 1 Å [fixed] in eight directions) due to light irradiation.

TEM results and deformation by light irradiation

The TEM images are shown in Figure S2. The images are slightly blurry (with a visible lattice fringe) because of the vibration of the sample holder connected to the optical fibre. The diffraction patterns from this particle are shown in Figure S3. To confirm atomic transfer under light irradiation, the intensity difference of the diffraction patterns between the light-on–light-off conditions were calculated. Similar calculations were carried out for the simulated diffraction patterns, and the results were compared. The best-matched results and their atomic arrangements are shown in Figure S3. They

did not match the difference results exactly; however, the diffraction pattern differences between the light-off and light-on conditions could be attributed to the movement of Cu atoms.

Cu_T displacement and diffraction pattern intensity

The differential patterns between the diffraction pattern of the original Cu_T position (light-off in the case of the experiment) and that of Cu_T displacement (light-on in case of the experiment) were compared to search for the best candidate for Cu_T displacement in the experiment. The diffraction patterns depending on Cu_T positions were simulated using ReciPro, a comprehensive crystal analysis software.[S3] The diffraction pattern simulations were carried out for sixteen cases in which Cu_T atoms were set at a constant distance from the original position and with a π/8 step from the a-axis. The $1\ \bar{3}\ 0$ and $2\ \bar{3}\ 0$ spots were mentioned in the main text, and the 1 1 0 and its equivalent spots, indicated with black arrowheads in the right column of Figure S4, were observed for comparison. It was found that the displacement of Cu_T caused the focused spots intensity to change, but the optimum Cu_T displacement could not be determined from the difference. From the results, one example of the atomic arrangement and diffraction patterns is shown in Figure S4.

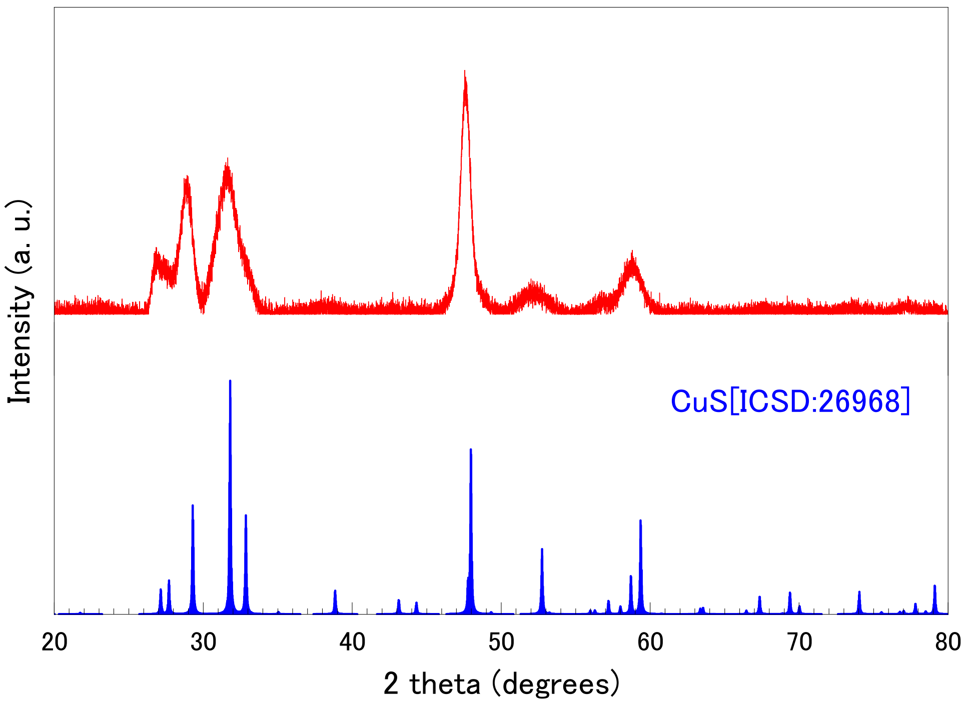

Figure S1. XRD pattern of CuS NCs.

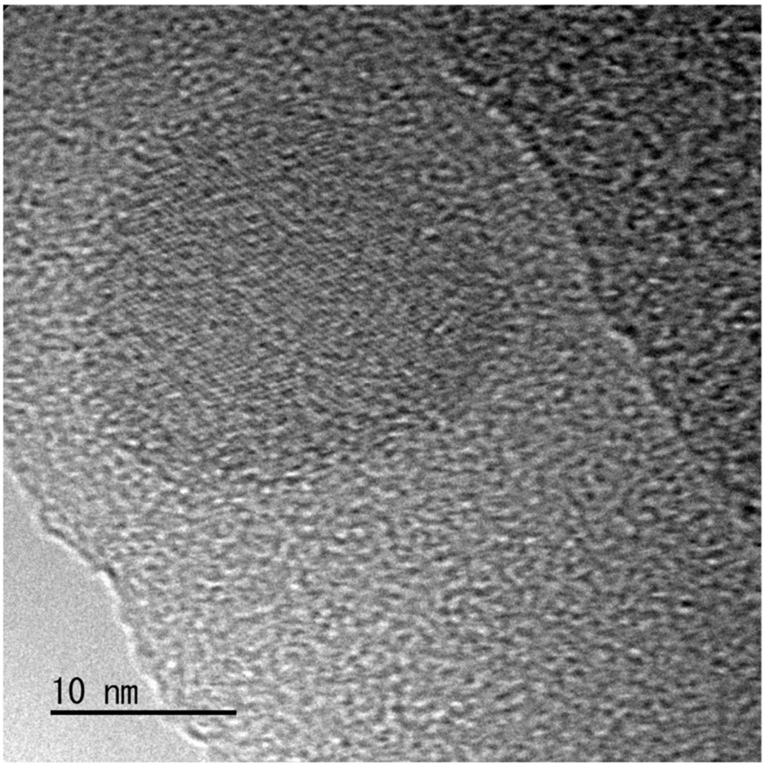

Figure S2. HRTEM image. The CuS NC is on the edge of the support, and the lattice fringe is visible.

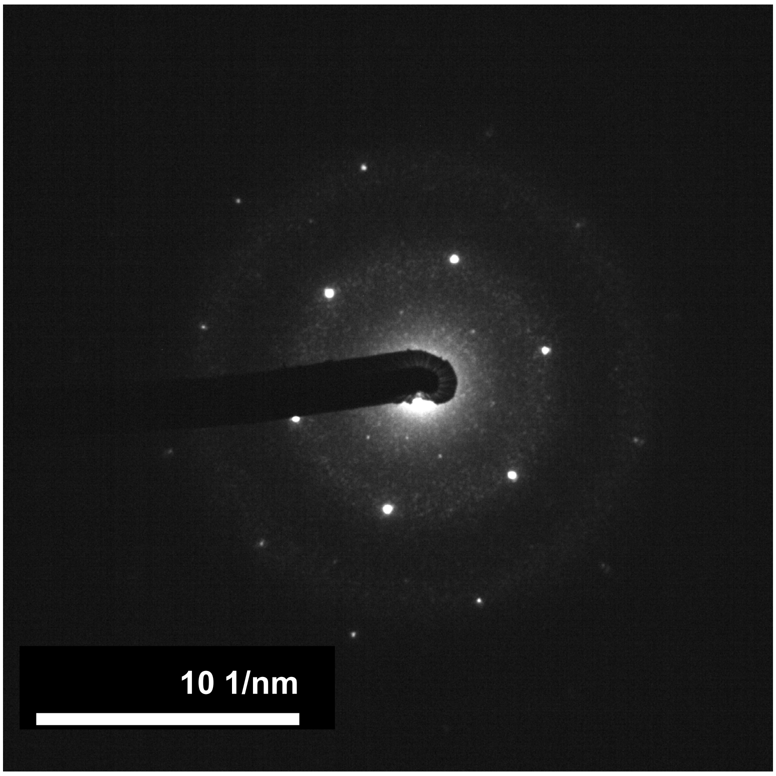

Figure S3. Electron-diffraction pattern of CuS without light illumination.

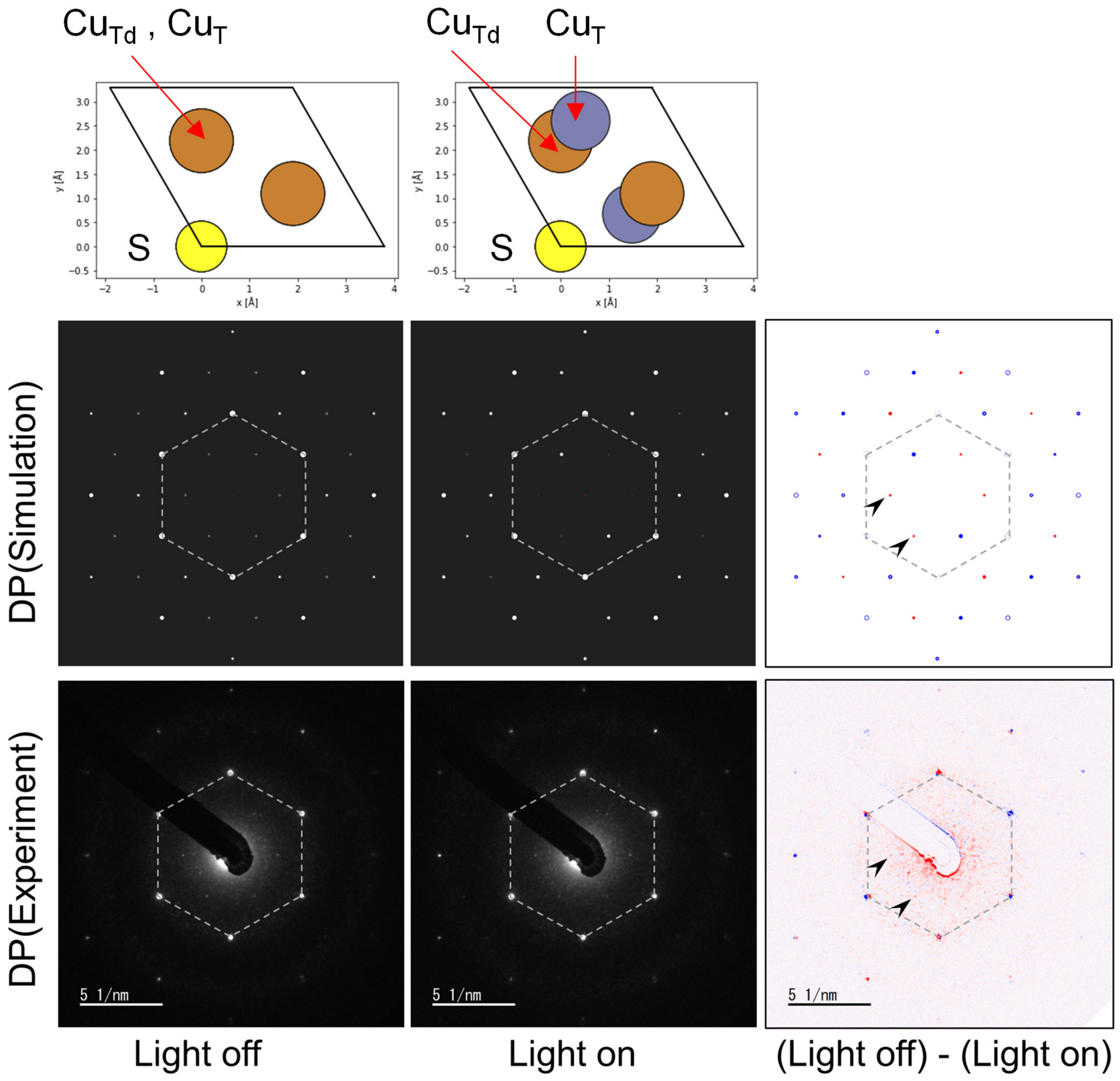

Figure S4. Atomic arrangements and diffraction patterns. The upper row shows atomic arrangements. The c-axis lies vertical to the plane of the paper, and the atomic movement has been magnified in these figures for easier understanding. The middle and lower rows show the simulated and experimental results, respectively. The left and middle columns show the light-on and -off conditions, respectively, while the right column shows their differences. In the differences, black arrowheads have been added as highlights to indicate spots where the experimental and simulation results vary.

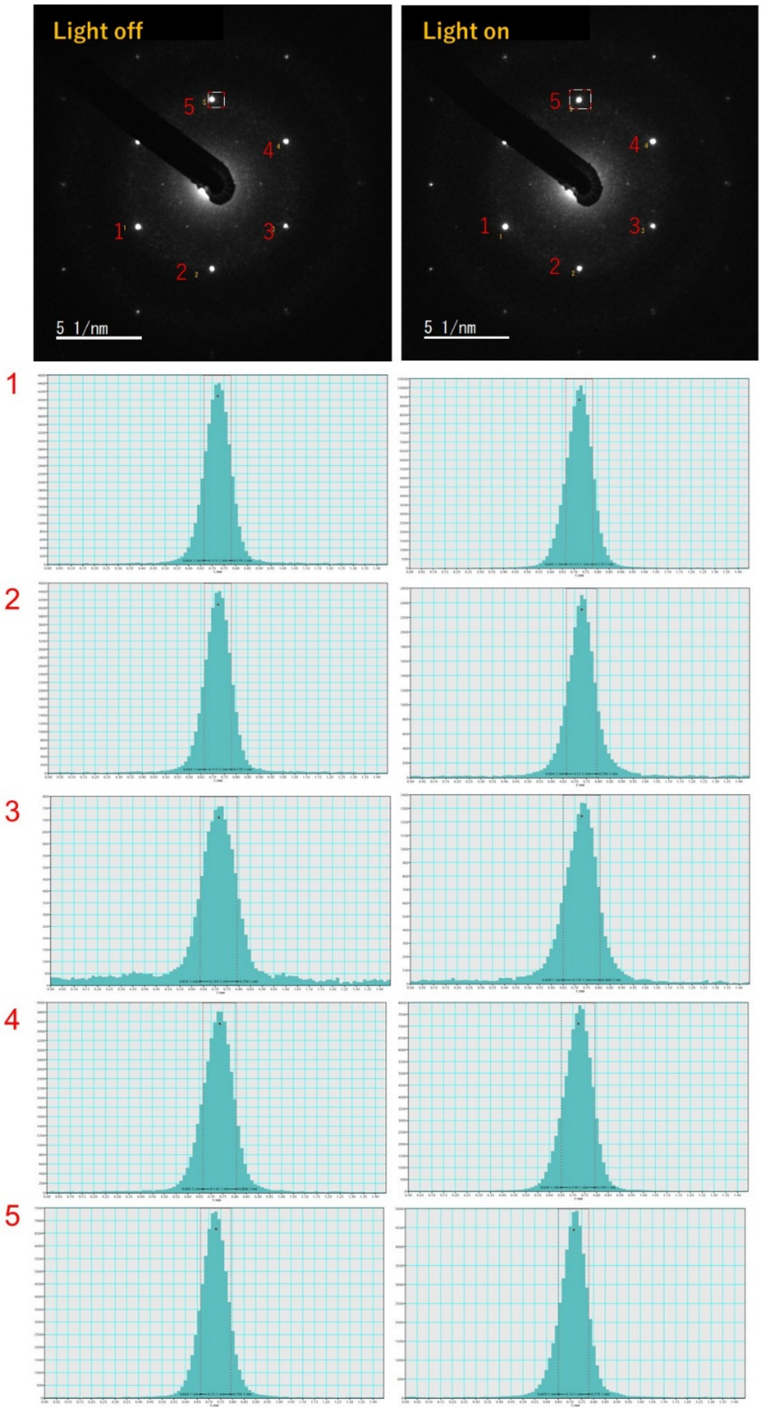

	FWHM [1/nm]	
Point	Light off	Light on
1	0.115	0.115
2	0.115	0.13
3	0.159	0.159
4	0.144	0.144
5	0.13	0.13
average	0.1326 ± 0.00832	0.1356 ± 0.00743

Figure S5. Full width at half maximum (FWHM) of diffraction spots with and without light illumination.

Estimation of temperature increase by laser heating

To clarify the photo-thermal effect on the CuS NCs by the 400- and 800-nm light, we estimated the temperature rise caused by the incident light. Using the fluence of incident light (F, 5 mJ cm^{-2}), absorptivity (A), film thickness (d, 60 nm), specific heat (c, 0.5 J K^{-1} g^{-1})[1], and density (ρ, 4.64 g cm^{-3}) of CuS NCs, the temperature rise (T) induced by the 400- and 800-nm light were calculated to be 30 and 11 K, respectively using the following equation:

$$FA = cT\rho d$$

where the absorptivity is 3.2% for 800 nm and 8.5% for 400 nm, as shown in Figure S6.

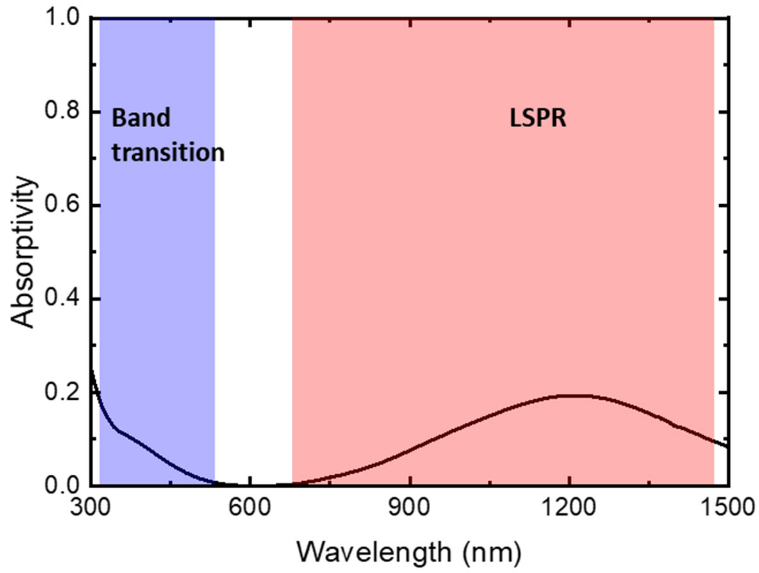

Figure S6. Absorptivity of CuS NCs in the thin film. Blue and red coloured areas show the band transition and LSPR absorption, respectively.

The diffraction intensity change due to temperature rise can be estimated using the Debye–Waller factor of the diffraction intensity; however, the Debye–Waller B-factors of CuS in the covellite structure have not been reported. Therefore, we estimated the diffraction intensity change due to temperature rise using the mean square displacement of the atoms from the linear compressibility and atomic distance of CuS. Because the atoms in a crystal randomly vibrate with a force constant (k), the mean square displacement (U) of atoms is expressed as follows[S4]

$$U = (\Delta x_{\mathrm{rms}})^2 = \frac{k_{\mathrm{B}}T}{2k}$$

where, k_{B} is the Boltzmann constant. The force constant can be derived from the linear compressibility (K, 3.77×10^{12} 1/Pa)[S5] and atomic distance (a, 2.19 Å) as

$$k = \frac{a}{K}$$

The Debye–Waller B-factor (B) and Debye–Waller factor of the 1 1 0 diffraction intensity ($D_{1\,1\,0}$) were calculated from the mean square displacement as

$$B(T) = 8\pi^2 U(T)$$

$$D_{1\,1\,0}(T) = \exp(-B(T) \cdot {q_{1\,1\,0}}^2)$$

where $q_{1\,1\,0}$ is the scattering vector from the (110) plane. In the kinematic theory of diffraction, the diffraction intensity (I) is proportional to the square of the Debye–Waller factor as

$$I(T) \propto |D_{1\,1\,0}(T)|^2.$$

Therefore, the intensity change (ΔI) due to temperature rise (11–30 K) is estimated to be 0.14–0.38% indicated by,

$$\Delta I = 1 - \frac{I(T)}{I(T=293\ \mathrm{K})}.$$

Thus, it was confirmed that simple photo-thermal effects did not cause photoinduced changes in the diffraction pattern of CuS NCs.

The Debye–Waller analyses were based on bulk CuS, and the experimentally obtained values for the Debye–Waller effect (Figure S7) are more appropriate for estimating the rise in temperature of CuS NCs by photoexcitation. According to the fitting curve in Figure S7c, the intensity changes due to temperature rises of 11 and 30 °C, corresponding to 0.44% and 1.2%, respectively, are still negligible.

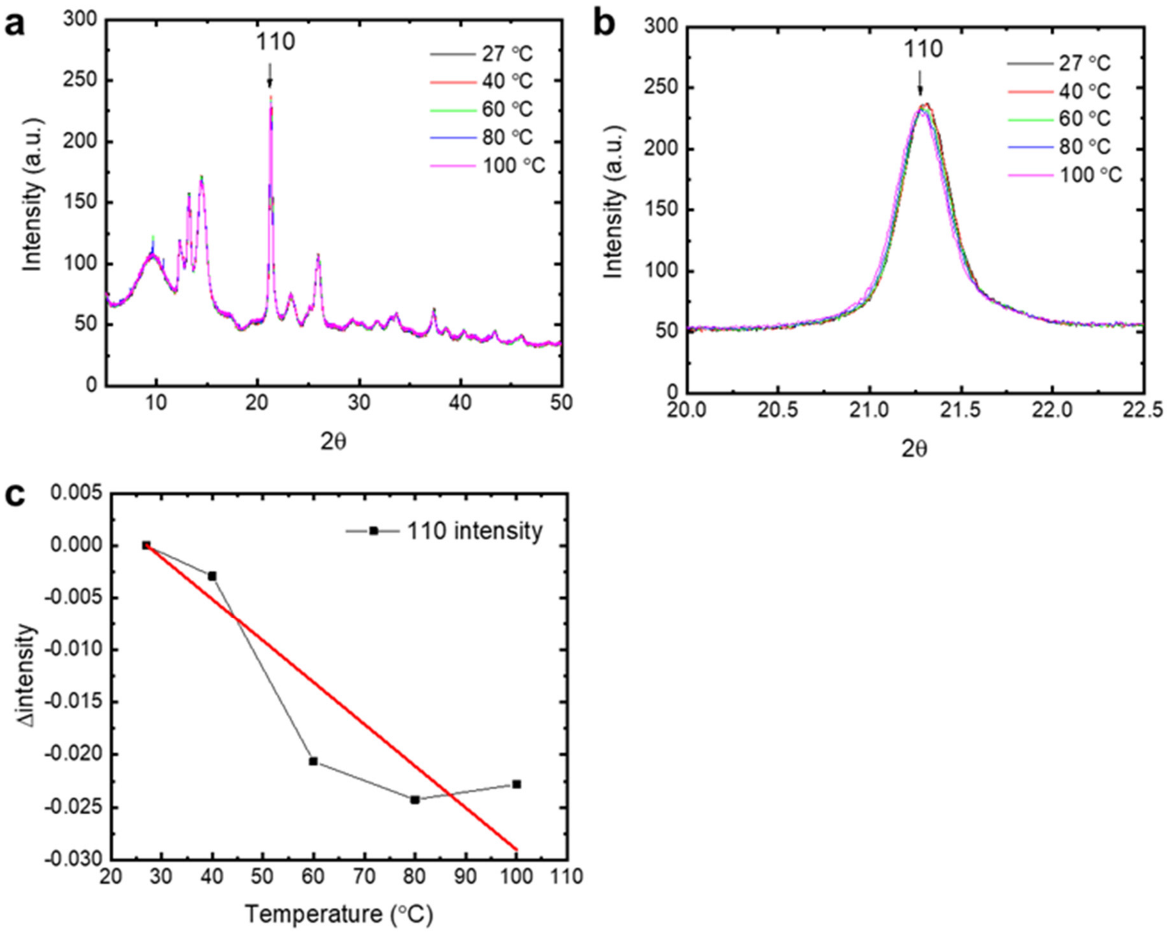

Figure S7. Estimation of the photo-thermal effects on CuS NCs by measuring the temperature dependence of X-ray diffraction patterns at the Aichi Synchrotron Radiation Center (Beam line: BL5S2). The X-ray wavelength of the synchrotron radiation was 0.7 Å. **(a)** X-ray diffraction patterns at various temperatures. **(b)** Enlarged view of the X-ray diffraction patterns at the (110) plane, shown by black arrows. **(c)** Peak intensity shifts as a function of temperature.

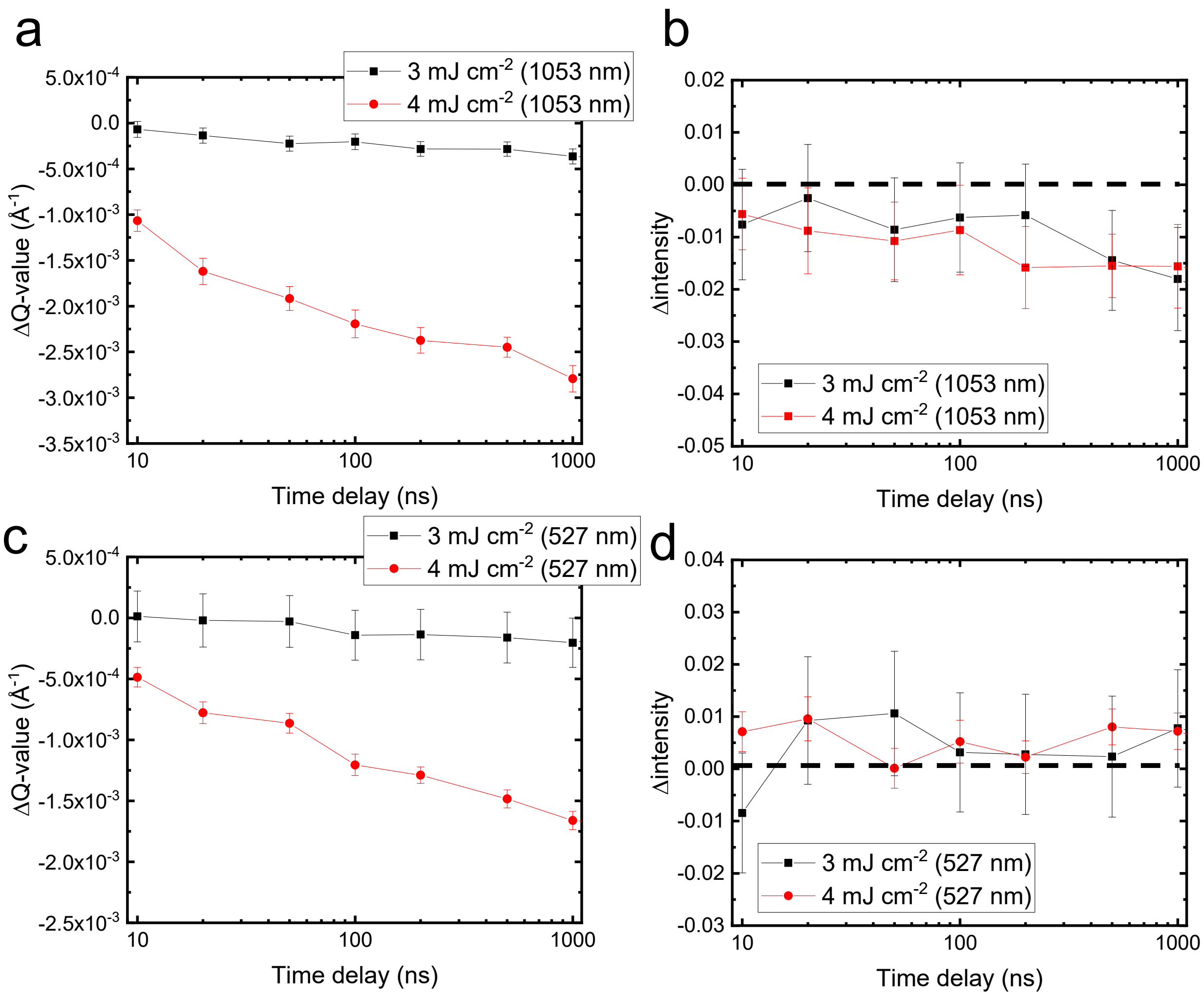

Figure S8. The excitation wavelength or fluence dependent shift in the *Q*-value from the (110) planes under ns-laser excitation. **(a)** Time evolution of the *Q*-value from the (110) planes under ns-laser excitation at a wavelength of 1053 nm. **(b)** Time evolution of the electron diffraction intensity from the (110) planes under ns-laser excitation at a wavelength of 1053 nm. The dotted line indicates zero. The negative Δintensity indicates a laser-induced shift in the intensity resulting from the ionic displacement at a ns-laser excitation wavelength of 1053 nm. **(c)** Time evolution of the *Q*-value from the (110) planes under ns-laser excitation at a wavelength of 527 nm. **(d)** Time evolution of the electron diffraction intensity from the (110) planes under ns-laser excitation at a wavelength of 527 nm. The dotted line indicates zero. The Δintensity was almost zero, indicating that no ionic displacement occurred at a ns-laser excitation of 527 nm. The error bars represent the standard deviation at each time delay.

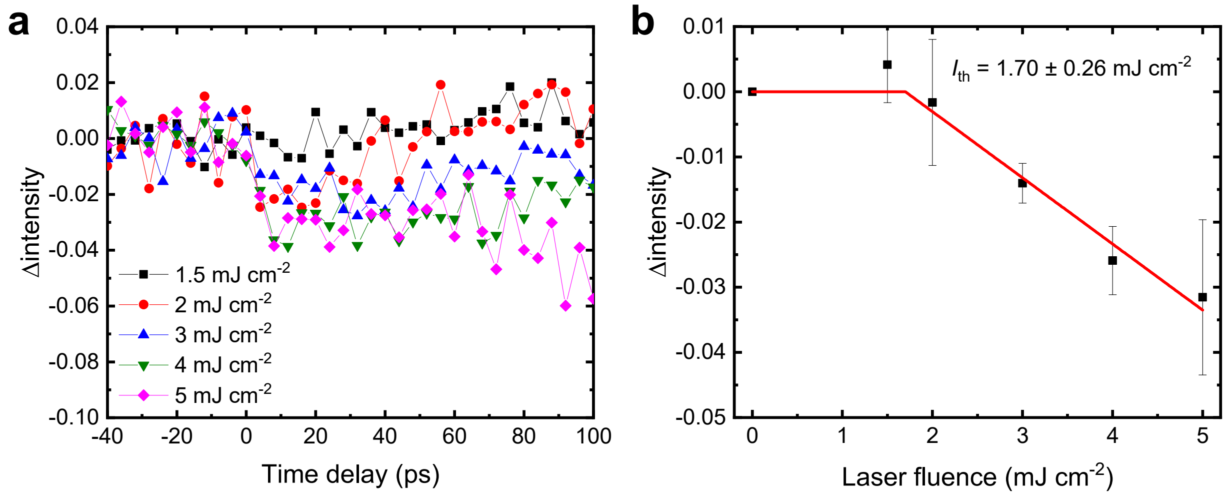

Figure S9. Laser fluence-dependent shift in the electron diffraction intensity from the (110) planes. (a) Time evolution of electron diffraction intensity from the (110) planes under fs-laser excitation with different fluences at a wavelength of 800 nm. (b) Laser fluence-dependent shift in the electron diffraction intensity from the (110) planes. The error bars were derived from the standard deviation at the time delay of 0–100 ps.

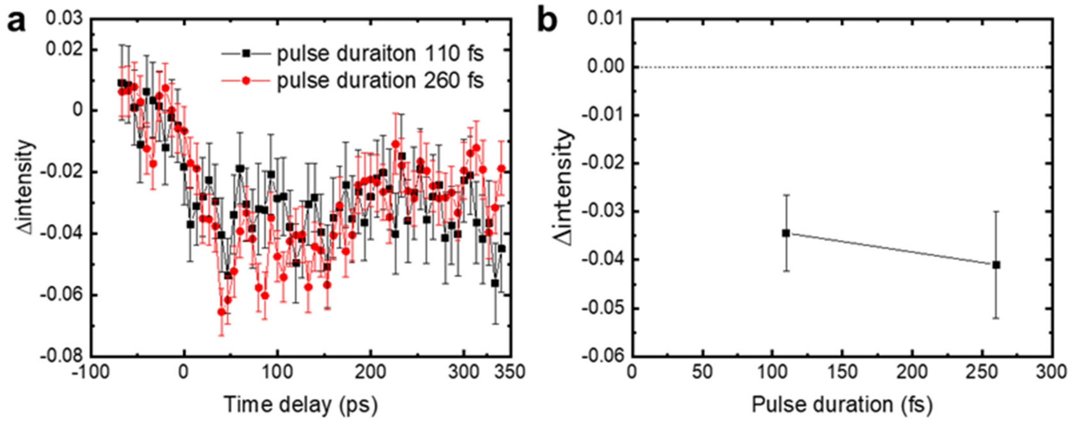

Figure S10. Pulse durations dependent shift in the electron diffraction intensity from the (110) planes under fs-laser excitation. (a) Time evolution of the electron diffraction intensity from the (110) planes under fs-laser excitation (wavelength: 800 nm, fluence: 5 mJ cm^{-2}) under different pulse durations. For the time-resolved electron diffraction experiments at a pulse duration of 260 fs, we introduced N-BK7 media (length of 200 mm) in the pump arm to extend the pulse duration by dispersion. The error bars represent the standard deviation at each time delay. (b) Intensity shifts as a function of pump pulse duration derived from (a). The intensity shifts at the pulse durations of 110 and 260 fs for the pump pulse are identical, which suggests that multiphoton absorption does not occur in this fluence range. This result is also consistent with the fact that the intensity shifts in the electron diffraction intensity are changed linearly by the laser fluence (Figure S9). The error bars were derived from the standard deviation at the time delay of 100–200 ps.

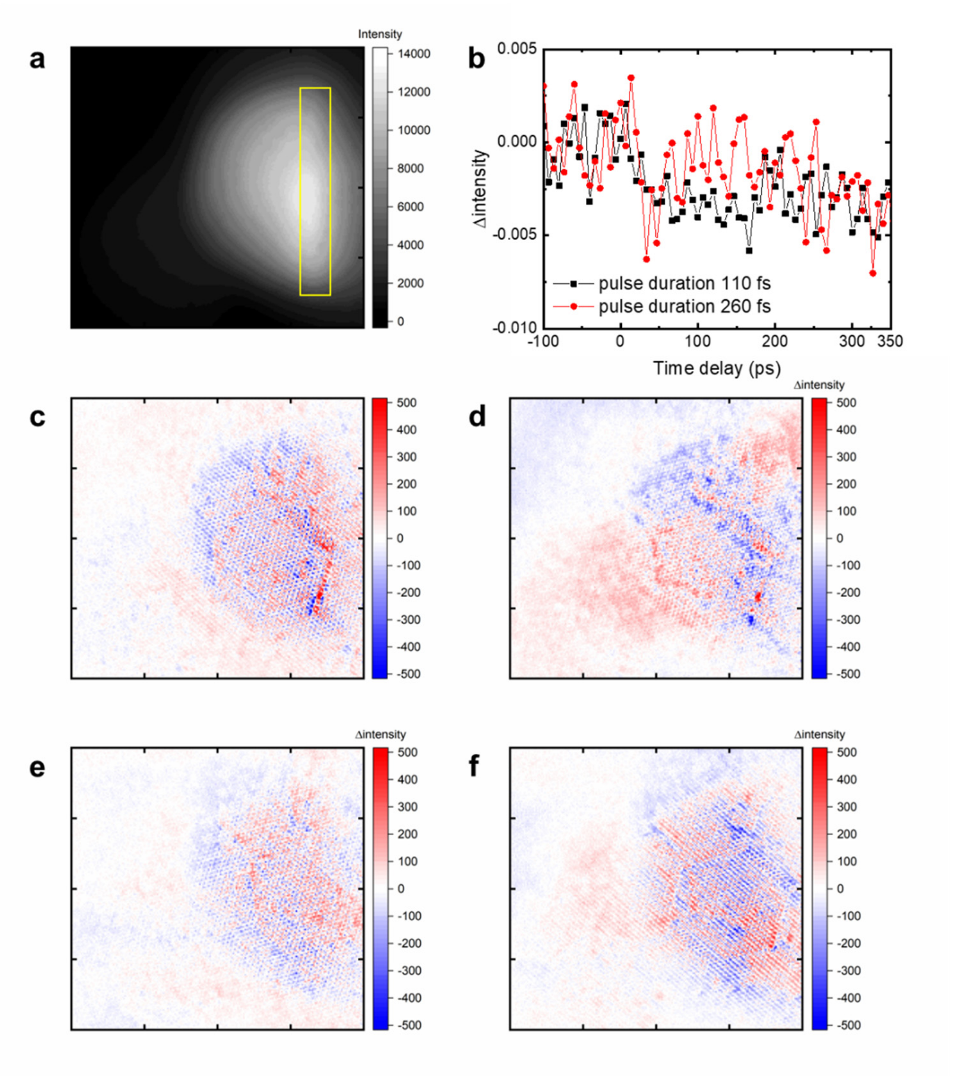

Figure S11. Observation of photoemission from the CuS sample under the fs-laser excitation. **(a)** Electron image for observation of photoemission from the sample surface. The CuS sample was set parallel to the electron beam. The sample cut half the electron beam (in diameter), and half of the electron beam travelled through and was detected by the CCD camera downstream. The wavelength and fluence of the pump optical pulse were fixed at 800 nm and 5 mJ cm^{-2}, respectively. **(b)** Time evolution of electron intensity changes on surface area. The electron intensity decreases slightly (~0.4%) after $t = 0$ due to scattering via the LSPR effect. The scattering does not change by the pulse duration, indicating that multiphoton absorption does not occur in this fluence range. The differential images at -20 ps **(c)** and +40 ps **(d)** at the pulse duration of 110 fs for the pump pulse and at -20 ps **(e)** and +40 ps **(f)** at the pulse duration of 260 fs. The slight decreases on the surface area and slight increases at the position away from the surface were observed after $t = 0$ for the pump pulse durations of 110 fs **(d)** and 260 fs **(f).**

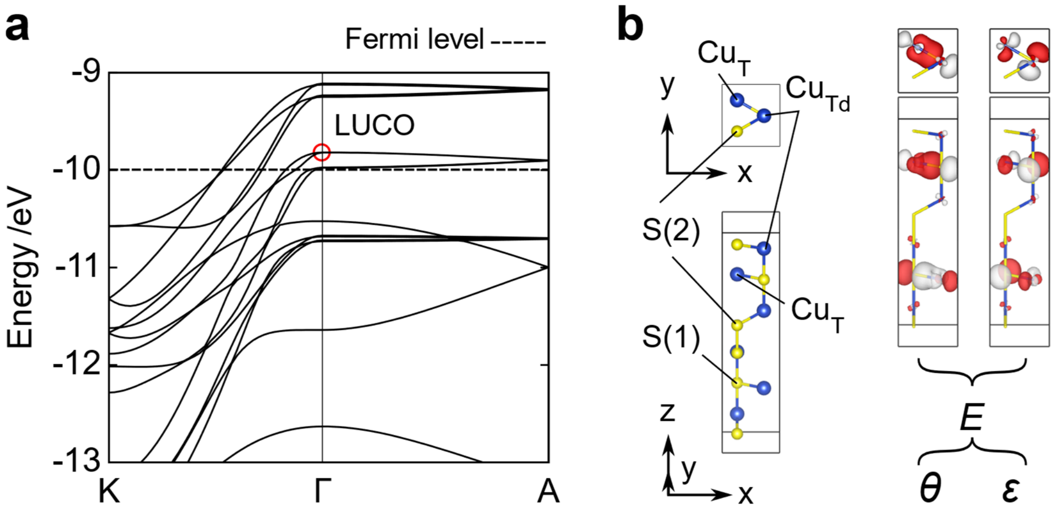

Figure S12. Theoretical calculations of band structure and lowest unoccupied degenerate crystal orbital (LUCO) of CuS. (a) Enlarged view of the calculated band structure of CuS. (b) Lowest unoccupied degenerate crystal orbital (LUCO) at the Γ point, where θ and ε represent lines for doubly degenerate representation E. For the top view of the atoms and crystal orbitals, only the upper half of them in the z-direction are shown for clarity. Isosurface values of the crystal orbitals are 5.0×10^{-2} a.u.

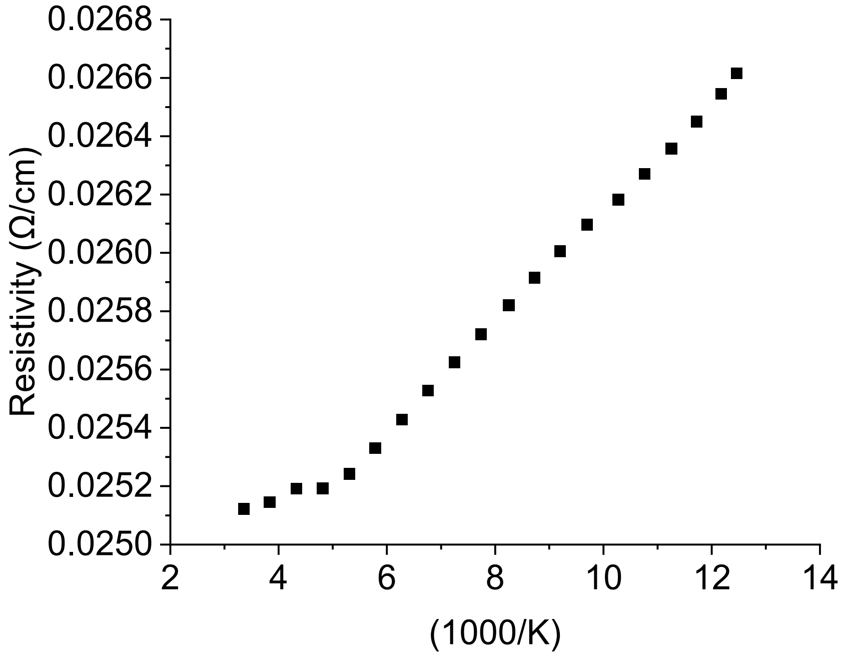

Figure S13. Temperature-dependent resistivity of the CuS film. Because the resistivity decreased with increasing temperature, the CuS film exhibited semiconductive behaviour. It confirmed that the rise in temperature caused by IR irradiation did not decrease conductivity.

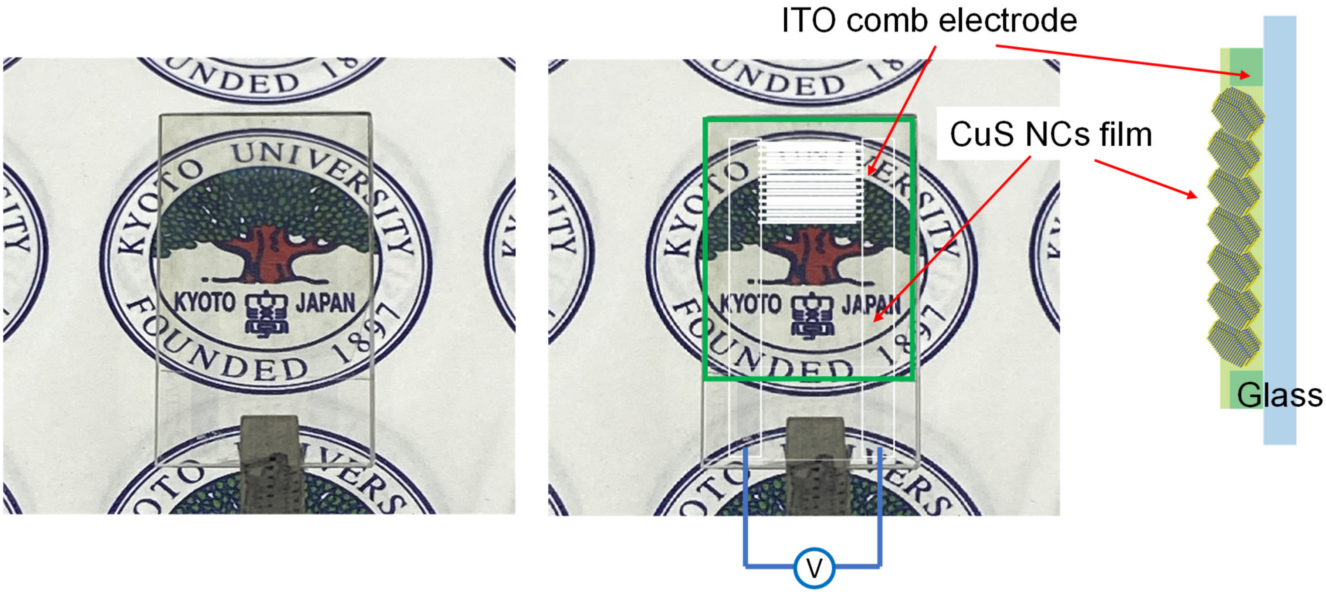

Figure S14. Photograph and schematic illustration of transparent plasmonic IR sensor. CuS film was deposited on the surface of the ITO comb electrode on glass. Because both the CuS NC film and ITO are transparent, the fabricated IR sensing device is transparent as well.

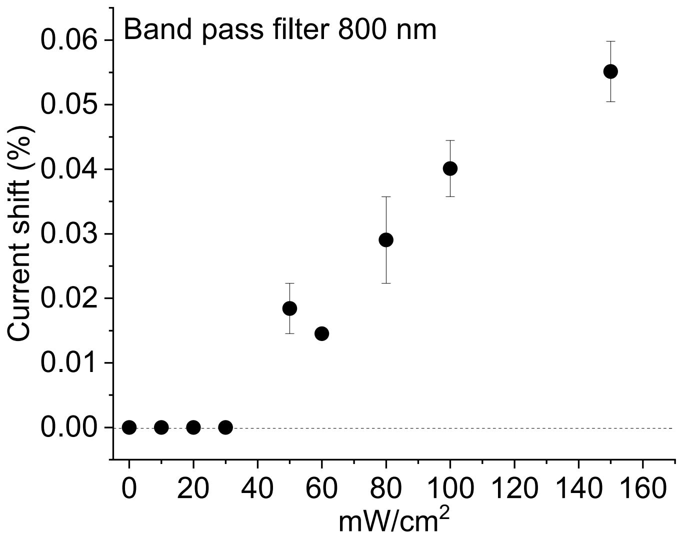

Figure S15. Power dependence of continuous-wave IR light (800 nm, Xe lamp)-responsive photoconductivity of the CuS NC film at a voltage of +1 V.

References

[S1] Xing, J., Takeguchi, M., Hashimoto, A., Cao, J. & Ye, J.. *Appl. Phys. Lett.* **104,** 163105 (2014).

[S2] The abTEM code: transmission electron microscopy from first principles; https://abtem.readthedocs.io/en/latest/index.html#.

[S3] Seto, Y. & Ohtsuka, M. *J. Appl. Cryst.* **55,** 397–410 (2022).

[S4] Hada, M., *et al. J. Chem. Phys.* **145,** 024504 (2016).

[S5] Selivanov, E. N., Gulyaeva, R. I. & Vershinin, A. D. *Inorg. Mater.* **43,** 573–578 (2007).

[S6] Saida, Y., *et al. J. Phys. Chem. A* **126**, 6301–6308 (2022).
